# Magnesium Levels in Drinking Water and Coronary Heart Disease Mortality Risk: A Meta-Analysis

**DOI:** 10.3390/nu8010005

**Published:** 2016-01-02

**Authors:** Lei Jiang, Pengcheng He, Jiyan Chen, Yong Liu, Dehui Liu, Genggeng Qin, Ning Tan

**Affiliations:** 1 Department of Cardiology, Guangdong Cardiovascular Institute, Guangdong General Hospital, Guangdong Academy of Medical Sciences, Guangzhou 510000, China; leijiang223@sina.com (L.J.); pengchenghe223@163.com (P.H.); jiyanchen888@yeah.net (J.C.); yongliu223@sina.com (Y.L.); dehuiliu123@sina.com (D.L.); 2 Department of Radiology, Nanfang Hospital, Southern Medical University, Guangzhou 510000, China; genggengqin123@163.com

**Keywords:** magnesium, coronary heart disease, mortality, meta-analysis

## Abstract

Epidemiological studies have demonstrated inconsistent associations between drinking water magnesium levels and risk of mortality from coronary heart disease (CHD); thus, a meta-analysis was performed to assess the association between them. Relevant studies were searched by the databases of Cochrane, EMBASE, PubMed and Web of Knowledge. Pooled relative risks (RR) with their 95% CI were calculated to assess this association using a random-effects model. Finally, nine articles with 10 studies involving 77,821 CHD cases were used in this study. Our results revealed an inverse association between drinking water magnesium level and CHD mortality (RR = 0.89, 95% CI = 0.79–0.99, *I*^2^ = 70.6). Nine of the 10 studies came from Europe, and the association was significant between drinking water magnesium level and the risk of CHD mortality (RR = 0.83, 95% CI = 0.69–0.98). In conclusion, drinking water magnesium level was significantly inversely associated with CHD mortality.

## 1. Introduction

Coronary heart disease (CHD) killed almost seven million people worldwide in 2010, accounting for the largest fraction of death causes and years of life lost [1] and may be the main cause of disease burden worldwide by 2020 [2], thus, primary prevention of CHD is of significant concern. In 1979, an association between water hardness (hardness of drinking water is largely determined by its calcium and magnesium content) and the risk of CHD mortality was reported [3].

It has previously been reported that drinking magnesium-rich water may be beneficial [4]. A prior study also suggested that the daily intake of magnesium is lower in developed countries [5]. Magnesium deficiency has been shown to predispose to cardiac arrhythmias through a variety of mechanisms [6]. Postmortem studies of people who suddenly died from CHD have found significantly lower levels of magnesium in uninfarcted heart muscle [7]. The evidence suggests that levels of magnesium in drinking water may affect mortality, if not morbidity, due to CHD [4]. Up to now, many studies have been conducted to investigate the association between drinking water magnesium levels and CHD mortality risk, with inconsistent results. To our knowledge, there has been no comprehensive meta-analysis to assess this association; therefore, we conducted this study to see if any correlation exists between drinking water magnesium levels and CHD mortality.

## 2. Methods

### 2.1. Literature Search Strategies

The relevant articles were identified by searching the databases of Cochrane, EMBASE, PubMed and Web of Knowledge written in English. The following search strategy was carried out: (magnesium OR Mg OR drinking water) AND (coronary heart disease (CHD) OR myocardial infarction (MI) OR ischemic heart disease (IHD)) AND (mortality). The references list was also reviewed to include all the related articles.

### 2.2. Eligibility Criteria

The current analysis included all the observational studies which reported drinking water magnesium levels and CHD mortality risk. The outcome measure was the incidence of the mortality of CHD or IHD or MI. The exposure of interest was drinking water magnesium levels. All included studies provided the relative risks (RR) or odds ratio (OR) and their 95% confidence intervals (CI), or provided enough data to calculate them. If the articles werefrom the same study or same populations, we then included only the most recent study; reviews or letters to the editor were excluded.

### 2.3. Data Extraction

All papers that were chosen based on the detailed search strategy were read by two of the authors (Lei Jiang and Pengcheng He). The following information was taken from each study: the publication years, study type, geographic locations, sex, number of participants, the results of CHD mortality outcome and the adjustment factors. The RR estimates and 95% CI for drinking water magnesium levels and CHD mortality risk were also extracted. We extracted the multivariable RR and its 95% CI if possible. Otherwise, we abstracted the crude RR estimates.

### 2.4. Statistical Methods

The inverse variance-weighted mean of the logarithm of RR and their 95% CI was calculated to evaluate the association between drinking water magnesium levels and CHD mortality. A random-effects model was used to combine study-specific RR (95% CI) [8]. Heterogeneity was assessed by Cochran Q and *I^2^* statistics [9]. Meta-regression analysis and subgroup analysis (study type, geographic locations, CHD outcomes and sex) were conducted to explore the potentially important covariate on the high heterogeneity if possible [10]. The small study effect was tested by Egger’s test [11]. Sensitivity analysis [12] was performed for removal of an individual study if the pooled RR lay out of the 95% CI. All the analyses were performed by STATA version 10.0. A 2-tailed *p* value of <0.05 represented significance.

## 3. Results

### 3.1. Study Characteristics

**Figure 1:**
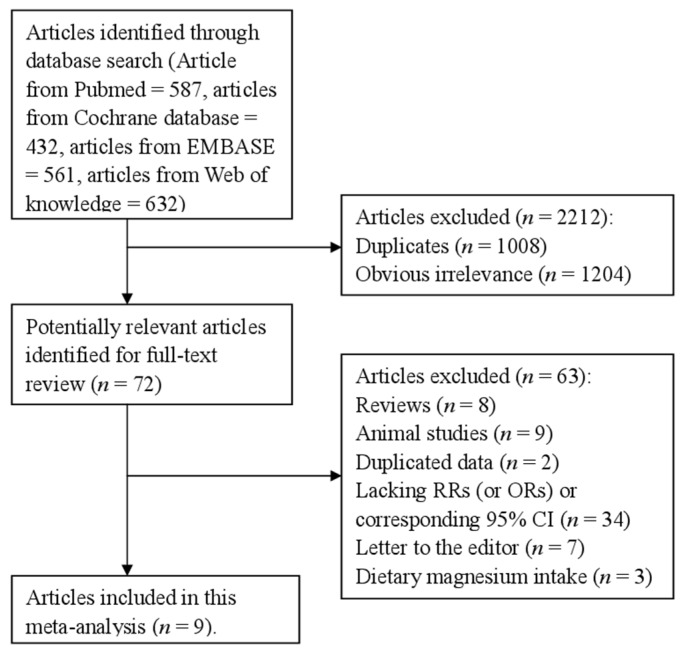
The flow diagram of screened, excluded, and analyzed publications.

Five hundred eighty-seven articles from PubMed, 432 articles from Cochrane database, 561 articles from EMBASE and 632 articles from the Web of Knowledge were selected by our search strategy. After reviewing the title/abstract, 72 articles were reviewed in full, 63 of which were subsequently excluded for various reasons. One paper reported the males and females respectively for the association between drinking water magnesium levels and the risk of CHD mortality [13]. Therefore, we put them as two separate studies. Finally, nine articles [3,13,14,15,16,17,18,19,20] involving 77,821 cases were included in this study. These articles included three cohort studies and seven retrospective studies. Figure 1 showed the flow diagram for our literature search. The characteristics of included studies are listed in Table 1. Four studies came from Sweden, two from Netherlands, two from Finland, one from England and one from China.

**Table 1:** Characteristics of studies on the levels of magnesium in drinking water and CHD mortality risk.

Study Year	Country	Study Design	Participants (Cases)	Age (Years)	CHD Outcome	Category (mg/L)	RR (95% CI) for Highest Versus Lowest Category	Adjustment for Covariates
Leurs *et al*. 2010	Netherlands	Prospective study	33,258 (1642)	55–69	IHD	Male 1.7–3.8 4.2–6.0 6.0–8.0 8.0–8.2 8.5–26.2 Female 1.7–3.8 4.2–6.0 6.0–8.0 8.0–8.2 8.5–26.2	Male 1 1.04(0.76–1.42) 1.21(0.87–1.71) 0.95(0.67–1.33) 1.23(0.82–1.86) Female 1 1.08(0.73–1.58) 0.75(0.49–1.18) 0.97(0.62–1.53) 0.89(0.50–1.59)	Adjusted for Age, current smoking, number of cigarettes smoked, years of active smoking, diabetes, hypertension, BMI, dietary calcium, dietary magnesium, saturated fat, monounsaturated fat, polyunsaturated fat, fruit and vegetable consumption, alcohol consumption, total energy intake (kilocalories), physical activity, educational level, volume of water consumption, magnesium or calcium concentration in tap water (depending on the exposure variable), use of diuretics, and use of multivitamins with minerals or calcium supplementation
Luoma *et al.* 1983	Finland	Case-control study	100 (50)	30–64	MI	Highest *vs.* lowest	1.63(0.62–4.52)	Adjusted for age and municipality with the cases.
Maheswaran *et al.* 1999	England	Case-control study	2,496,659 (64,226)	≥45	IHD	Highest *vs.* lowest	1.01 (0.96–1.05)	Adjusted for Age, sex, Carstairs deprivation quintile and geographical gradients.
Punsar *et al.* 1979	Finland	Prospective study	1711 (198)	40–59	CHD	Highest *vs.* lowest	0.64(0.44–0.91)	Na.
Rosenlund *et al.* 2005	Sweden	Case-control study	458 (116)	45–70	IHD	0.20–0.9 0.9–1.9 1.9–3.5 3.5–19.2	1 1.07(0.73–1.55) 0.86(0.59–1.26) 0.97 (0.66–1.41)	Adjusted for Age, sex, catchment area, smoking, hypertension, socioeconomy, job strain, diabetes mellitus, body mass index, and physical inactivity.
Rubenowitz *et al.* 1996	Sweden	Case-control study	1843 (854)	50–69	IHD	≤3.5 3.6–6.8 6.9–9.7 ≥9.8	1 0.88(0.66–1.16) 0.70(0.53–0.93) 0.65(0.50–0.84)	Adjusted for Age and magnesium and calcium, respectively.
Rubenowitz *et al.* 1999	Sweden	Case-control study	1746 (378)	50–69	IHD	≤3.4 3.5–6.7 6.8–9.8 ≥9.9	1 1.08(0.78–1.49) 0.93(0.64–1.34) 0.70 (0.50–0.99)	Adjusted for Age and magnesium and calcium, respectively.
Rubenowitz *et al.* 2000	Sweden	Case-control study	521 (263)	50–74	IHD	Highest *vs.* lowest	0.64 (0.42–0.97)	Adjusted for Age and magnesium and calcium, respectively.
Yang *et al.* 2006	China	Case-control study	20,188 (10,094)	50–69	IHD	≤7.7 7.8–13.5 14.1–41.3	1 1.00(0.93–1.08) 1.09 (0.99–1.19)	Adjusted for Age, sex, urbanization level of residence, and magnesium and calcium levels in drinking water respectively.

Abbreviations: CHD, coronary heart disease; IHD, ischemic heart disease; MI, myocardial infarction; BMI, body mass index; CI, confidence interval; RR, relative risk; Na, not available.

### 3.2. High versus Low Analyses

**Figure 2:**
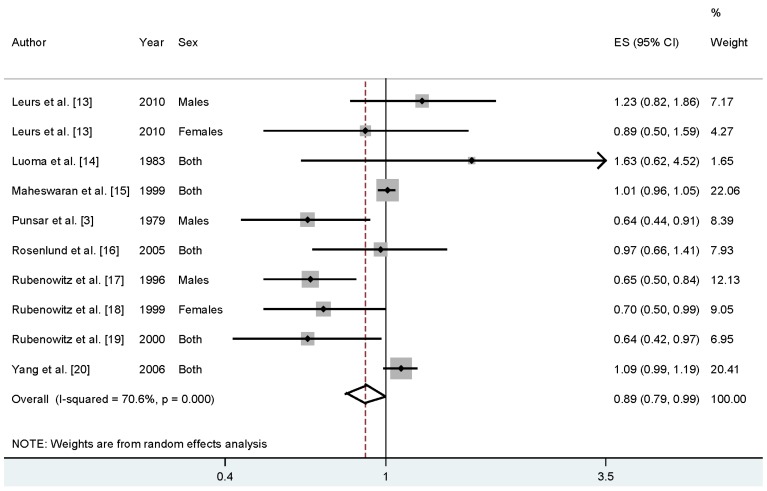
The forest plot between the levels of magnesium in drinking water and CHD mortality risk.

Four studies reported that higher drinking water magnesium levels may reduce the CHD mortality risk, while four studies reported an increase but nonsignificant association. When we pooled the overall results, the association was significant between drinking water magnesium levels and CHD mortality risk (RR = 0.89%, 95% CI = 0.79–0.99, *I*^2^ = 70.6%) (Figure 2).

### 3.3. Meta-Regression and Subgroup Analysis

In the pooled results, high heterogeneity (*I*^2^ = 70.6%, *P*_heterogeneity_ = 0.000) was found. Thus, we conducted univariate meta-regression to explore the cause of this high heterogeneity with the covariates of publication year, location, sex, study type, CHD mortality outcome, and number of cases. No significant association was found in the above covariates.

There were seven studies conducted in case-control design and three conducted in cohort design, and the RR was 0.87 for case-control studies (95% CI = 0.76–0.98). However, the association was nonsignificant in the cohort studies. In subgroup analyses for geographic locations, the highest levels of magnesium in drinking water versus the lowest levels were significantly associated with reduced risk of CHD mortality in Europe (summary RR = 0.83, 95% CI = 0.69–0.98). There were three studies that reported the association in males and two studies in females. However, no significant associations were found either in males or females. Furthermore, in stratified analysis by CHD mortality outcomes, the association was only significant in the MI group (RR = 0.81, 95% CI = 0.64–0.98). Detailed results for the overall and subgroup analyses are shown in Table 2.

**Table 2:** Summary risk estimates of the levels of magnesium in drinking water and CHD mortality risk.

Subgroups	No.	No.	RR (95% CI)	Heterogeneity Test	
	Cases	Studies		*I^2^* (%)	*p*-Value
All studies	77,821	10	0.89 (0.79–0.99)	70.6	0.000
Study design					
Cohort	1840	3	0.88 (0.57–1.34)	63.5	0.064
Case-control	75,981	7	0.87 (0.76–0.98)	74.6	0.001
Geographic locations					
Europe	67,727	9	0.83 (0.69–0.98)	70.0	0.001
Asia	10,094	1	--	--	--
CHD outcome					
IHD	65,868	3	1.01 (0.96–1.05)	0.0	0.586
MI	11,755	6	0.81 (0.64–0.98)	78.6	0.000
CHD	198	1	--	--	--
Sex					
Males	2194	3	0.78 (0.54–1.15)	73.3	0.024
Females	878	2	0.75 (0.56–1.00)	0.0	0.484

Abbreviations: CHD, coronary heart disease; IHD, ischemic heart disease; MI, myocardial infarction; CI, confidence interval; RR, relative risk.

### 3.4. Sensitivity Analysis and Small Study Effect

**Figure 3:**
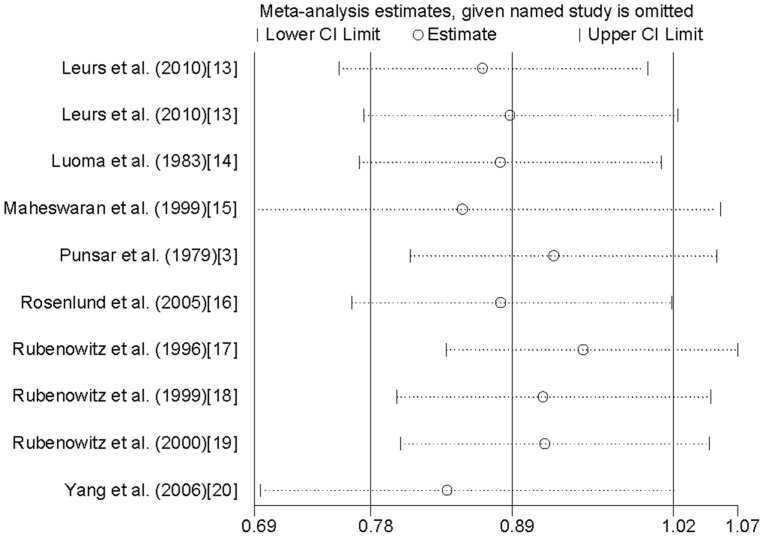
Analysis of influence of individual study on the association between the levels of magnesium in drinking water and CHD mortality risk.

Sensitivity analysis (Figure 3) did not identify any one individual study that strongly influenced the results on drinking water magnesium levels and CHD mortality risk publication bias between drinking water magnesium levels and CHD mortality was confirmed by Egger’s test (*p* = 0.475).

## 4. Discussion

We conducted the first comprehensive meta-analysis that looked at an association between drinking water magnesium levels and CHD mortality risk. The findings from this meta-analysis suggest that the high levels of magnesium in drinking water may reduce the risk of CHD mortality. The association was also found in case-control studies, as well as in the European population.

In our pooled results, we found evidence of high between-study heterogeneity. A previous study [21] had reported that between-study heterogeneity is common in meta-analyses. Therefore, meta-regression with publication year, location, sex, study type, CHD mortality outcome, and number of cases was used to explore potential covariates which might cause this high between-study heterogeneity. Unfortunately, using the above mentioned covariates, no significant association was found to be the cause of the high heterogeneity. Furthermore, we conducted the subgroup analysis by geographic location, study design, CHD outcomes and sex to explore the potential heterogeneity, and heterogeneities did arise in some subgroup analyses.

A highlight of this study is the large sample size (77,821 cases) we were able to utilize in finding an inverse association between drinking water magnesium levels and CHD mortality risk. But, there are also some limitations in our study. First, nine of the 10 studies were conducted in Europe, and only one study was in China. A significant association was found only when we pooled the results for the subgroup of European studies. Notably, six of the ten studies came from Scandinavia (Sweden and Finland). Therefore, the results may be more applicable to the European population, and especially to Scandinavian populations. More studies originating in other countries are required to assess this association. Second, our study included seven retrospective studies and three cohort studies. For retrospective studies, original studies could cause some recall or selection bias, but proper epidemiological method requires clarification of the association in the original article. In our results, the association was only significant in retrospective studies, but not in cohort studies. This is because only three studies included in this study were cohort design. Therefore, further studies with cohort design are required. Third, there are only few studies which report the association between the level of magnesium in drinking water and CHD mortality for males and females respectively. Although we pooled the results for the subgroup analysis by sex, the small sample size of studies lead to lower statistical power. Finally, we found evidence of high between-study heterogeneity in the pooled analysis and some subgroup analyses. However, meta-regression could not explain this high heterogeneity. Thus, some other genetic and environment variables may affect this high heterogeneity.

In conclusion, findings from this study showed that a higher level of magnesium in drinking water was significantly inversely associated with the risk of CHD mortality, especially among Scandinavian populations.
